# The cost-utility of early use of high-flow nasal cannula in bronchiolitis

**DOI:** 10.1186/s13561-021-00339-7

**Published:** 2021-10-28

**Authors:** Jefferson Antonio Buendía, Ranniery Acuña-Cordero, Carlos E. Rodriguez-Martinez

**Affiliations:** 1 Departamento de Farmacología y Toxicología, Facultad de Medicina, Grupo de Investigación en Farmacología y Toxicología, Universidad de Antioquia, Carrera 51D, #62-29 Medellín, Colombia; 2 Departamento de Neumología Pediátrica, Hospital Militar Central, Bogotá, Colombia; 3 Departamento de Pediatría, Facultad de Medicina, Universidad Militar Nueva Granada, Bogotá, Colombia; 4 Department of Pediatrics, School of Medicine, Universidad Nacional de Colombia, Bogotá, Colombia

**Keywords:** Health economics, Public health, Healthcare, Asthma, Oxygen, Cannula

## Abstract

**Background:**

High-flow nasal cannula (HFNC) oxygen is a non-invasive ventilation system that was introduced as an alternative to CPAP (continuous positive airway pressure), with a marked increase in its use in pediatric care settings. This study aimed to evaluate the cost-effectiveness of early use of HFNC compared to oxygen by nasal cannula in an infant with bronchiolitis in the emergency setting.

**Methods:**

A decision tree model was used to estimate the cost-effectiveness of HFNC compared with oxygen by nasal cannula (control strategy) in an infant with bronchiolitis in the emergency setting. Cost data were obtained from a retrospective study on bronchiolitis from tertiary centers in Rionegro, Colombia, while utilities were collected from the literature.

**Results:**

The QALYs per patient calculated in the base-case model were 0.9141 (95% CI 0.913–0.915) in the HFNC and 0.9105 (95% CI 0.910–0.911) in control group. The cost per patient was US$368 (95% CI US$ 323–411) in HFNC and US$441 (95% CI US$ 384–498) per patient in the control group.

**Conclusions:**

HFNC was cost-effective HFNC compared to oxygen by nasal cannula in an infant with bronchiolitis in the emergency setting. The use of this technology in emergency settings will allow a more efficient use of resources, especially in low-resource countries with high prevalence of bronchiolitis .

## Introduction

High-flow nasal cannula (HFNC) oxygen is a non-invasive ventilation system that was introduced as an alternative to CPAP (continuous positive airway pressure), with a marked increase in its use in pediatric care settings [1–3]. In children with bronchiolitis, HFNC has been used as an alternative to CPAP and mechanical ventilation, with adequate tolerance [4–7].

HFNC has been associated with: increases in patients’ functional residual capacity, reductions in the effects of oxygen dilution in trachea compared to NC (nasal cannula), dead space washout, and more compliance in relation to CPAP [8]. This physiological effect has been reflected in randomized clinical trials, with significantly lower rates of treatment failure compared to NC, with few severe adverse events or safety issues [9]. Despite this evidence and the frequent use of HFNC in the PICU (pediatric intensive care unit) setting, the economic impact of its use in the emergency setting has not yet been evaluated. It is precisely its use in the emergency setting where this system could have the greatest economic impact as a prior alternative to using CPAP or mechanical ventilation [10]. This would be significant, especially for hospitals in middle-income countries with scare health resources, and where this technology could be a cost-saving alternative [11, 12]. This study aims to evaluate the cost-effectiveness of HFNC compared to oxygen by nasal cannula in infants with bronchiolitis in the emergency setting.

## Methods

### Design

Cost-utility study that compared HFNC against oxygen by nasal cannula (control group) in infants with bronchiolitis in the emergency setting. The effectiveness outcome was the quality-adjusted life years (QALYs). The analysis was carried out from a societal perspective (including direct and indirect costs). The analytic horizon was an acute episode of bronchiolitis (six days) [3]. Given the short time horizon, no type of discount to costs or results was applied. The study protocol was reviewed and approved by the Institutional Review Board of Clinica Somer (No 281015) and the University of Antioquia (No 18/2015).

### Economic model

**Fig. 1:**
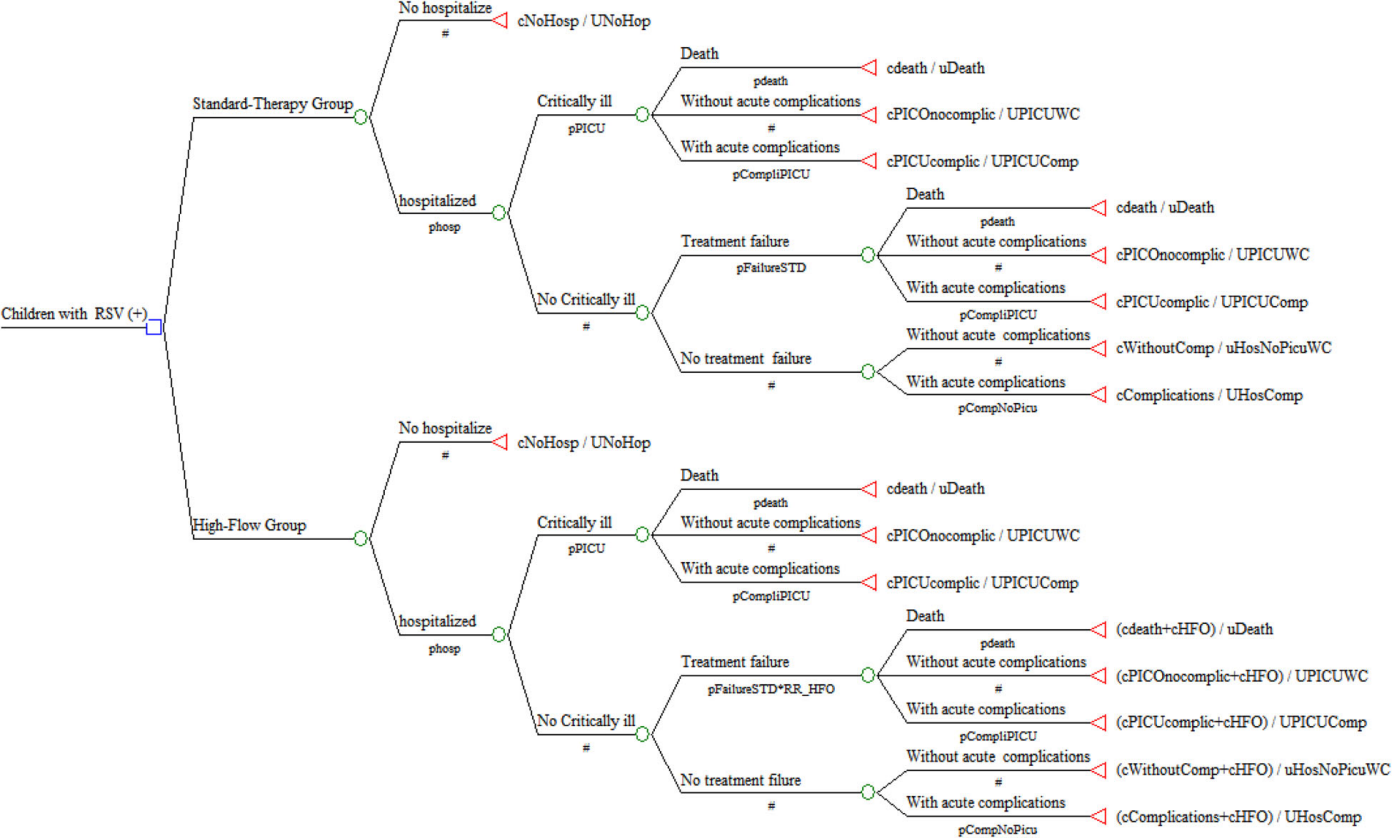
Decision tree model with probabilities estimated by outcome

A decision tree model was used to estimate the cost-effectiveness of HFNC on bronchiolitis (Fig. 1). In the economic model, we defined the following outcomes according to the natural history of bronchiolitis: death, hospitalization with or without acute complications, PICU admission with or without acute complications. Acute complications included: pneumonia, atelectasis, sepsis, pleural effusions, and pneumothoraxes [13, 14].

### Probabilities

To estimate the probabilities of the model (see Table 1), we performed a systematic review of systematic reviews or RCTs published or observational studies up to January 2020. To identify potentially relevant studies, we carried out searches of computerized databases (MEDLINE, CENTRAL, LILACS, and CINAHL) using the following search strategy: (High flow nasal cannula OR Nasal Cannula OR Nasal Cannulae OR oxygen) AND (Bronchiolitis OR Bronchiolitis, Viral), limited with the terms children OR child OR pediatric OR adolescents OR infants OR preschoolers). No language restrictions were applied. To be included in the model, the studies had to be parallel-group or cross-over RCTs, systematic reviews or RCTs published or observational studies including children between 2 and 18 years of age. Other observational clinical studies obtained during the review of references cited in the published literature were also included [15–22]. The computerized search yielded 927 citations and a total of 55 studies were examined in full for possible inclusion. For data to be included in the model, participants in the studies needed to be under the age of 18 years with bronchiolitis and studies needed to evaluate the use of high flow nasal cannula and report at least one of the following outcomes: percentage of hospitalizations or admissions in pediatric intensive care units or acute complications during the period of observation.

**Table 1:** Model inputs: morbidity probabilities used in base case and sensitivity analyses

Model input	Base case value	SA range for one-way sensitivity analyses	Source
**Probability**			
Hospitalization	0,25	0,01-0,41	(21)
PICU, given hospitalization	0,07	0,06-0,18	(20)
Mortality, given PICU admission	0,009	0,001–0,06	(16)
Acute complications, given hospitalization	0,13	0,10–0,20	(15)
Acute complications, given PICU admission	0,15	0,15-0,53	(23)
Treatment failure in control group	0.22	0.19–0.29	(25)
**Utility**			
No hospitalization	0,95	1,00–0,76	(31–34)
Hospitalization without acute complications	0,88	1,00–0,70	(35, 36)
Hospitalization with acute complications	0,59	0,70–0,47	(35, 36)
PICU without acute complications	0,73	0,87-0,58	(35, 36)
PICU with acute complications	0,51	0,60–0,40	(35, 36)
**HFNC effectiveness**			
Relative risk of reduction of treatment failure	0,57	0,45-0,72	(25)

### Interventions

Information regarding the effect of HFNC was extracted from a recent randomized clinical trial included after a systematic review, as discussed above, that compared HFNC with oxygen by nasal cannula (control group) in infants with bronchiolitis in the emergency setting [23]. This clinical trial included infants with bronchiolitis in the emergency department or inpatient unit if they needed supplemental oxygen to keep oxygen saturation in the range of 92–98%, and excluded critically ill infants who had an immediate need for ICU admission or had cyanotic heart disease, a basal skull fracture, upper airway obstruction or a craniofacial malformation and infants who were receiving oxygen therapy at home. Infants in this study were randomized and received humidified high flow oxygen at a rate of 2 l per kilogram of body weight per minute. Infants in the standard group received supplemental oxygen through a nasal cannula up to a maximum of 2 l per minute.

### Cost analysis

All costs and information on resource use were collected directly from the medical invoices of all patients hospitalized with a bronchiolitis diagnosis (ICD-10 code: J21.0) in tertiary centers in Rionegro, Colombia, between January 2018 and December 2018 (*n* = 416), Table 2. This cost and clinical characteristics of these patients were published previously [22]. Brief, the direct costs considered in the analysis include medical consultation at the emergency room, specialist referrals, chest physiotherapy, diagnosis support (laboratory, electrocardiogram, x-ray, etc.), medication (oxygen, nebulization, antibiotics, corticosteroids, bronchodilators, etc.), medical devices, hotel services in the intensive care unit, hotel services and overhead cost in the general medical ward. All treatment costs include the administration and preparation costs covered by the treating organization. All adverse events were assumed to be fully reversible and thus not to cause any additional costs to the hospital district. To avoid data errors during medical record abstraction, we used software (Excel MS®) with automatic calculation functions and error alerts and a review of outliers by the research team. We used US dollars (currency rate: US$ 1.00 = COP$ 3000) [24, 25] to express all costs in the study. For the valuation of the indirect costs associated with the loss of parents’ productivity, the human capital method was used, assuming everyone receives an income of at least a legal minimum wage for formal or informal work. The cost-opportunity of the productivity loss at the workplace and the caregiver was assessed based on the minimum wage without including transportation assistance (US$ 229.81 per month). The legal minimum wage approved by the government was taken as a reference and not an average or median wage thereof, given that in Colombia, over 75% of the population has this value as their income [26]. Because all patients with acute asthma episodes included in this study were children, we assumed that at least one family member accompanied the patient permanently during hospitalization, as pediatric hospitals in the country usually allow only one companion per patient in the hospital. The cost associated with transportation and food (does not include a stay), was assumed to correspond to 50% of minimum wage per day.

**Table 2:** Cost used in base case and sensitivity analyses

Model input	Base case value	SA range for one-way sensitivity analyses	Distribution γ(SD)
**Intervention cost**			
HFNC per patient day	58.19	50.2–61.5	(58.19)
**Hospitalization cost**			
Daily cost in pediatric ward	48.82	47,64 50.00	(3, 20)
Hospital length of stay (days)	5,8	4,00-6,01	(2,03)
**PICU related cost**			
Daily cost in PICU	327,35	326,26–328-43	(5,49)
PICU lenght of stay (days)	10	9,01-15,05	(3,08)
**Emergency visit prior hospitalization cost**			
Daily cost of emergency ward	12,83	12,19-13,46	(3, 20)
**Direct medical cost per patient-day**			
Specialist referrals	10,67	10,31-11,01	(1,72)
Chest physiotherapy	5,15	4,90-5,39	(1, 23)
Chest radiography	2,84	2,70-2,98	(0,73)
Others diagnostic imaging	0,01	0,0-0,022	(0,08)
Complete blood cell counts	1,12	1,05-1,17	(0,28)
RSV test	2,71	2,83-3,03	(2,72)
Other laboratory tests	4,40	4,23-4,47	(0,37)
Oxygen	1,37	1,28-1,45	(0,41)
Nebulization	16,23	1,28-1,45	(4,52)
LEV	1,10	1,07-1,13	(0,16)
Antibiotics systemic	1,21	1,11-1,30	(0,49)
Systemic o Inhaled Corticosteroids	0,08	0,0-0,90	(4, 18)
Bronchodilators	0,04	0,03-0,04	(0,02)
Other drugs	0,65	0,60–0,68	(0,04)
Medical devices	10,24	9,71-10,76	(2,66)
**Indirect cost patient-day**	9,24	6.38–18,07	(4, 30)

### Utilities

The utility values used for the QALY calculations were taken from the literature from the aforementioned systematic review. The utility value for hospitalization was 0.95 [27–30], whereas the utility value for PICU, given hospitalization, was 0.88 [31, 32]. The utility value for hospitalization with acute complications was 0.59; it was 0.5 for PICU with acute complications [33, 34]. The number of QALYs was calculated as the utility value given to a particular health state multiplied by the length of time spent in that state. Given that these utilities were extracted from studies in non-Colombian populations, a range was used for a one-way and probabilistic sensitivity analysis of around 20% of the utitity’s value.

### Sensitivity analyses

The robustness of the economic model was evaluated with one-way sensitivity analyses and probabilistic sensitivity analyses in accordance with the recommendation of consolidated health economics evaluation reporting standards [35]. Tornado diagrams were used as a graphical method for displaying one-way sensitivity analyses. Probabilistic sensitivity analyses were carried out using the Monte Carlo technique with a simulation of a hypothetical cohort of 10,000 patients, in which each parameter varied randomly according to certain distributions (beta distribution in the case of probabilities and gamma distribution in the case of costs) to generate expected cost utilities with 95% confidence intervals (95% CI). A cost-effectiveness acceptability curve was used to evaluate the uncertainty surrounding the cost-effectiveness of HFNC. We estimated the population expected value of perfect information to inform the expected cost of uncertainty (expected opportunity loss surrounding the decision) [36]. Microsoft Exel® was used in all analyses.

## Results

**Fig. 2:**
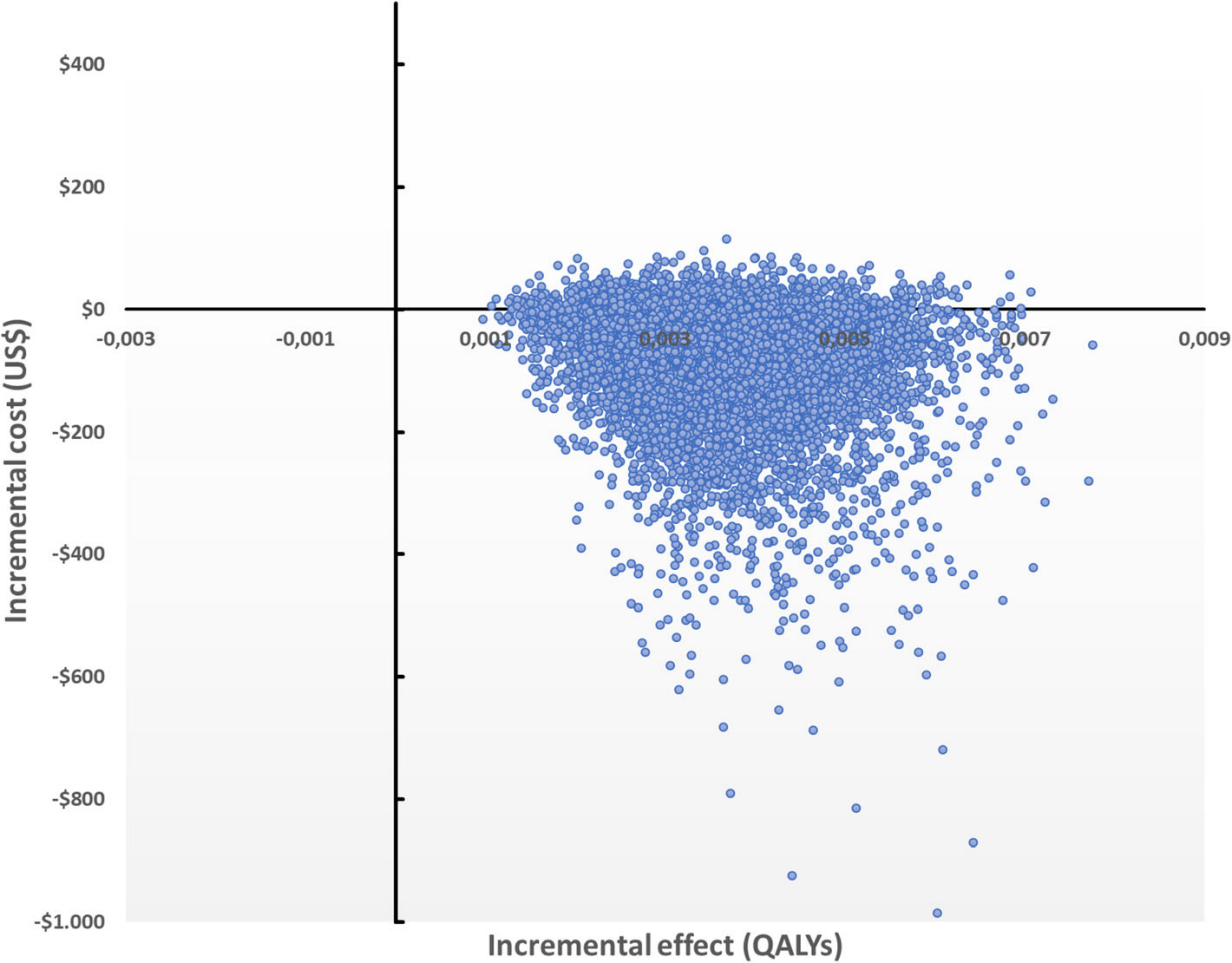
Cost effectiveness plane

The calculed QALYs per patient were 0.9141 (95% CI 0.913–0.915) in the HFNC group and 0.9105 (95% CI 0.910–0.911) in the control group. The expected cost per patient was US$ 368 (95% CI US$ 323–411) for HFNC and US$ 441 (95% CI US$ 384–498) for the control group per patient. The strategy control was dominated by HFNC with a negative incremental cost-effectiveness ratio, Table 3. The cost-effectiveness plane is presented in Fig. 2.

**Table 3:** Cost- effectiveness of HS nebulized vs Control group

Strategy	Cost (US$)	Difference	QALYs	Difference	Cost/QALY	ICER
HFNC	$ 368,89		0,91		403,55	
Control	$ 441,37	−72.46	0,91	0,004	484,75	(Dominated)

(HFNC) High-flow nasal cannula

ICER (Incremental cost-effectiveness ratio)

### Sensitivity analysis

**Fig. 3:**
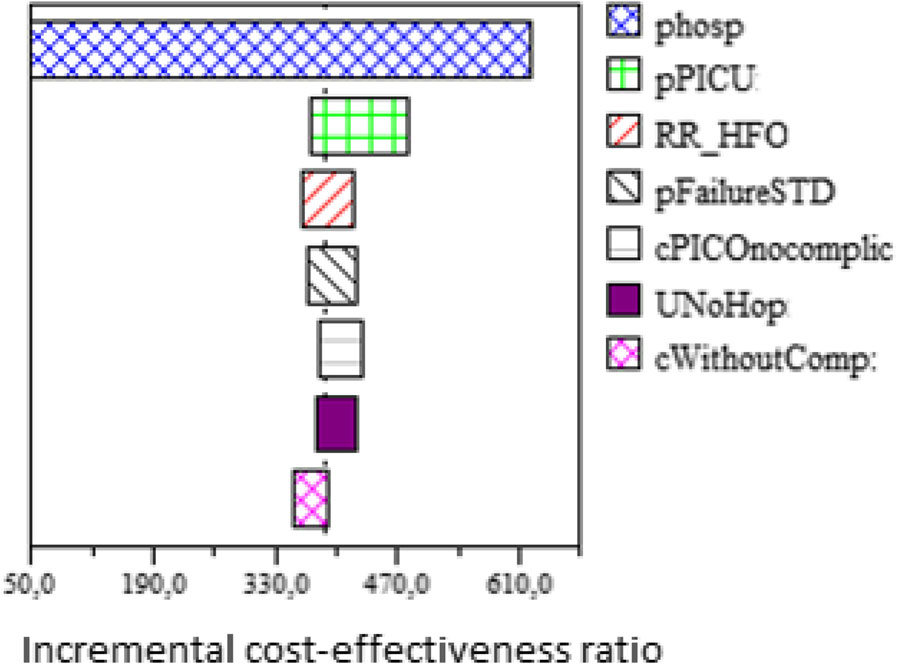
Tornado diagram. Phos:probability of hospitalization. pPICU: probability of PICU admission, given hospitalization. RR_HFO: Relative risk of reduction of hospitalization of High flow nasal cannula. cWithoutComp: cost of hospitalization without acute complication. UNoHop: utility of patient no hospitalized. pFailureSTD: probability of treatment failure in control group. cPICOnocomplic: cost of PICU admission without acute complications

**Fig. 4:**
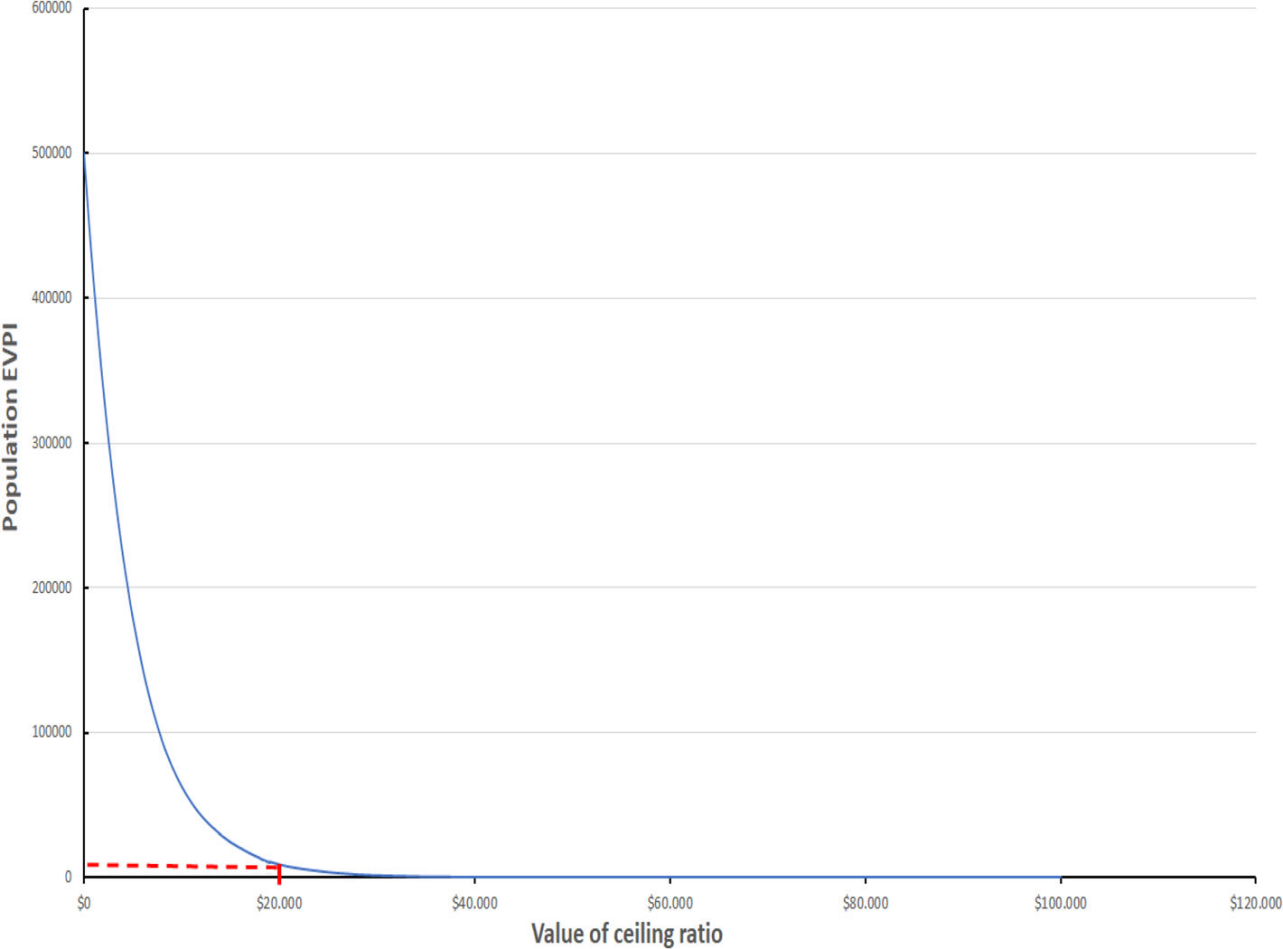
Population EVPI. *In red EVPI (US$ 19,000) for a Colombian threshold (3 times GDP per capita)

Sensitivity analyses of parameters showed that the cost-effectiveness of HFNC was sensitive to the probability of hospitalization, Fig. 3, without any threshold having been identified. For the entire range of this probability, a higher expected value was consistently detected in the HFNC strategy over the control group. Likewise, HFNC was always the cost-effectiveness strategy for all ranges of thresholds, Fig. 3. The population EVPI for a threshold of US$ 20,000 was US$ 19,000, Fig. 4.

## Discussion

Our study suggests that nebulized HFNC is unequivocally cost-effective and dominant over the control strategy, achieving better outcomes at a lower cost in hospitalized patients with bronchiolitis. The potential magnitude of cost savings for the health system (US$ 72 per patient), especially in a middle-income country, is not negligible if we consider that in our country we have around 95,000 annual cases of children under 2 years old being hospitalized with acute respiratory infections [21]. Our findings provide inputs for changes in clinical practice guidelines in similar settings, where efficiency in the allocation of health resources can be maximized.

Common concerns regarding the adoption of technologies relate to safety and effectiveness. Current evidence is conclusive regarding the safety of HFNC. In a recent systematic review of seven RTCs, no severe adverse events and no increased risk of air leak syndromes were reported [9]. Only mild skin lesions were reported in under 3% of patients. In addition, parents and carers favored HFNC because of the ability to feed and overall comfort [9, 37]. This aspect differs from CPAP, which is usually not well tolerated and interferes with patients’ normal care, and is associated with a higher risk of air leak syndromes [38]. In terms of HFNC’s effectiveness, the evidence is still debatable, with studies for and against its use concerning CN or CPAP in children with moderate or severe bronchiolitis both existing. The HFNC has been demonstrated to have had a beneficial effect on treatment failure, but many authors criticize this effect for the absence of effects on other related outcomes. For example, only one clinical trial included in the previously mentioned systematic review [39] found an effect on LOS and the duration of oxygen therapy, and in none of the trials included was there an effect on the probability of PICU transfer. It is clear that there was no impact in this trial on the risk of admission to PICU because all patients with CN failure went onto HFNC, while those with HFNC failure went directly to PICU in these studies. This therefore underestimated the impact on this outcome and affected the difference in hospital stay rates and the duration of oxygen therapy. Indeed, over 60% of patients with a failure of standard oxygen therapy that switched to HFNC successfully recuperated [23, 37].

There is clearly a need for more clinical trials with standardized oxygen weaning protocols, but this therapy currently constitutes an optimal resource prior to mechanical ventilation. While our study showed only a slight effect on utilities between the two strategies evaluated, due to the fact that HFNC does not directly impact the chance of complications, HFNC has a significant positive impact on the cost because it reduces the use of secondary resources, especially those arising from PICU admission. These findings arose from a previous economic study carried out in parallel during a clinical trial, demonstrating that the HFNC arm required fewer resources than the CN arm [37]. This is particularly important in scenarios with more limited economic resources. In at least four observational studies carried out in developing countries, a significantly lower proportion of children were transferred to PICU before the initial HFNC in emergency settings [5, 7, 40, 41]. Indeed, clinical trials published until now with larger sample sizes show that the magnitude of the effect of HFNC was higher in hospitals without on-site PICUs than in hospitals with on-site PICUs. This escalation of care occurred at a rate of 7% in the HFNC group compared to 28% in the standard therapy group, while in hospitals with on-site PICUs this endpoint occurred at 14% in the HFNC group and 20% in the standard therapy group [23]. Without a doubt, and reinforced by our results, the use of HFNC in emergency settings is an efficient alternative for infants with moderate-severe bronchiolitis to optimize the cost in scenarios without on-site PICUs or with limited economical resources.

A very important aspect of our model is that it was robust to changing the model’s utility and cost values. HFNC was always the most cost-effective strategy in all ranges of thresholds evaluated with a low population EVPI. This was consistent with the finding that, although our utilities were collected from other populations, our results did not change when exploring the change in the ICER in the range of values of each utility explored. The same happens with costs. Although the resources, frequencies of use, and costs were collected from tertiary centers in Rionegro and not from a national study of all hospitals in Colombia, modifications to their values in the sensitivity analysis also did not significantly change the ICER. These aspects give us confidence in relation to the ability to make decisions based on our results. As is always necessary in science, more studies are needed to replicate our results [42]. Our study has some limitations. The cost data were collected retrospectively. Bronchiolitis treatment and the costs in question, including hospital prices, did not markedly change. Furthermore, our country has been characterized by having very low price variation in the last 10 years, especially in terms of health services [24]. Additionally, we use utilities extracted from the literature and not estimated directly from our population. As was mentioned previously, the reliability and robustness of the results were evaluated using sensitivity analyses. However, an additional strength is the perspective of the society on which the economic analysis was focused, which allows a faster transfer of results to health policies.

In conclusion, HFNC in emergency settings was cost-effective for the hospital treatment of infants with moderate or severe bronchiolitis. The use of this technology in emergency departments will allow a more efficient use of resources, especially in low-resource settings with a high prevalence of respiratory diseases. Our study provides evidence that should be used by decision-makers to improve clinical practice guidelines and should be replicated to validate their results in other countries.

## Data Availability

The raw data supporting your findings can be request to CIEMTO (https:// http://ciemto.medicinaudea.co/).
